# *Toxoplasma gondii* Genotypes Circulating in Serbia—Insight into the Population Structure and Diversity of the Species in Southeastern Europe, a Region of Intercontinental Strain Exchange

**DOI:** 10.3390/microorganisms9122526

**Published:** 2021-12-07

**Authors:** Aleksandra Uzelac, Ivana Klun, Vladimir Ćirković, Neda Bauman, Branko Bobić, Tijana Štajner, Jelena Srbljanović, Olivera Lijeskić, Olgica Djurković-Djaković

**Affiliations:** Group for Microbiology and Parasitology, Center of Excellence for Food- and Vector-Borne Zoonoses, Institute for Medical Research, University of Belgrade, National Institute of Republic of Serbia, 11129 Belgrade, Serbia; aleksandra.uzelac@imi.bg.ac.rs (A.U.); iklun@imi.bg.ac.rs (I.K.); vladimir.cirkovic@imi.bg.ac.rs (V.Ć.); neda.bauman@imi.bg.ac.rs (N.B.); bobicb@imi.bg.ac.rs (B.B.); tijana.stajner@imi.bg.ac.rs (T.Š.); jelena.srbljanovic@imi.bg.ac.rs (J.S.); olivera.lijeskic@imi.bg.ac.rs (O.L.)

**Keywords:** *Toxoplasma gondii*, population, structure, genotypes, Africa, Europe

## Abstract

In Europe, *Toxoplasma gondii* lineage II is dominant, and ToxoDB#1 the most frequently occurring genotype. The abundance of lineage III genotypes varies geographically and lineage I are rare, yet present in several regions of the continent. Data on the *T. gondii* population structure in southeastern Europe (SEE) are scarce, yet necessary to appreciate the diversity of the species in Europe. To help fill this gap, we genotyped 67 strains from nine species of intermediate hosts in Serbia by MnPCR-RFLP, determined the population structure, and identified the genotypes using ToxoDB. A neighbor-joining tree was also constructed from the isolates genotyped on nine loci. While 42% of the total genotype population consisted of ToxoDB#1 and ToxoDB#2, variant genotypes of both lineages comprised 46% of the population in wildlife and 28% in domestic animals and humans. One genotype of Africa 4 lineage was detected in a human sample. Interestingly, the findings include one lineage III variant and one II/III recombinant isolate with intercontinental distribution, which appear to be moderately related to South American genotypes. Based on these findings, SEE is a region of underappreciated *T. gondii* genetic diversity and possible strain exchange between Europe and Africa.

## 1. Introduction

The Apicomplexan protozoan *Toxoplasma gondii* has been ranked as the fourth food and waterborne parasite of global importance in terms of public health concerns out of 24 candidate organisms, and the first among protozoan agents [1]. In a similar exercise in Europe a few years later, *T. gondii* was ranked second, only after *Echinococcus multilocularis,* so again as the first protozoan [2]. *T. gondii* has three infectious life stages—the tachyzoite, bradyzoite, and sporozoite—all of which are transmissible, either from host to host (tachyzoite and bradyzoite) or from the environment to the host (sporozoite). The dominant mode of horizontal transmission is via ingestion of bradyzoites in tissue cysts or sporozoites in oocysts [3]. Transfer of tissue cysts via donor organs into naive recipients, or extremely rarely of tachyzoites via donor blood, is also possible [4,5]. Tachyzoites can also be vertically (transplacentally) transmitted from the mother to the fetus [4,5]. Major transmission vehicles are meat, as bearers of encysted bradyzoites, and water, which if contaminated with sporulated oocysts, can cause epidemic outbreaks [3,6,7,8]. Soil contaminated with oocysts may be another vehicle for infection for grazers and birds who feed off the ground, while oocysts in both water and soil can also contaminate vegetables and fruits and thus enter the foodweb of humans and animals. *T. gondii* tachyzoites and bradyzoites replicate by endodyogeny, which allows transmission solely by shuttling between intermediate hosts, that is, all homeothermic species. Sexual reproduction of tachyzoites happens only in the gut of the Felidae, and therefore the definitive hosts, resulting in the formation of oocysts. Oocysts are excreted into the environment along with feces, where they sporulate in appropriate ambient conditions to reach full maturity and infectivity [9,10]. Sexual reproduction also leads to genome recombination and facilitates evolution of the species.

Clinical experience with acquired *T. gondii* infection in the human immunocompetent population worldwide shows that disease is most often associated with mild pathology, while the infection is mostly asymptomatic and without clearly discernible pathology [4], despite the fact that the natural progression of the infection ends in the development of tissue cysts that are predominantly localized in neurons and myocytes [3,4,5]. Nonetheless, severe cases have been reported sporadically, most often in South America, and in travelers returning from Central America, Asia, and Africa [11,12]. A plausible explanation of these clinical observations may be derived from the growing insight into the global population structure of *T. gondii*, which has revealed that different genotype populations circulate in different geographical areas [13]. In addition, it has been experimentally demonstrated that infection with a number of isolates from South America results in mortality of laboratory mice, which indicates virulence [14]. Lineages I, II, III and likely Africa 1 circulate globally, while lineages of apparent limited geographical distribution have been detected in North America [15], South America [16], and Africa [17,18]. Differences in the diversity of the genotype population not only appear to be geographical, but also related to the eco-system, that is, the anthropogenic versus the wild environment. This is evident in the highly diverse genotype population which characterizes the non-anthropogenic environment of the Amazon basin, while in North America it has been shown that the diversity of the species increases as the distance to the anthropogenic environment increases [19,20]. In contrast, the lower diversity of the strain population in North America and especially Europe is due to the overwhelmingly high frequency of archetype II (ToxoDB#1), while similarly, the high frequency of China 1 (ToxoDB#9) is responsible for the low diversity in China [13], both genotypes being of low virulence to mice [4,5,21].

However, diversity in much of the world still needs to be interpreted with caution, as the lion’s share of isolates originates from humans and domestic animals, and thus also makes up the majority in the ToxoDB (www.toxodb.org (accessed on 20 June 2021)) and other genotype collections. In fact, strains isolated from pigs, chickens, and sheep are over-represented, while wildlife strains are under-represented. Thus, the reported dichotomy between strain population structure and diversity circulating in the anthropogenic environment versus the sylvatic environment may also reflect the inherent strain origin bias [19,20].

Despite a formidable effort to isolate, genotype, and identify *T. gondii* strains circulating in various hosts residing in different environments in Europe, particularly in France, there are still major gaps in the data. *T. gondii* strains originating from wildlife species, such as predatory mammals and birds, have rarely been isolated and/or genotyped, indicating incomplete knowledge regarding the virulence spectrum of the genotype population in Europe. A major spatial gap also includes the region of southeastern Europe (SEE). Thus, to shed some light into the population structure of *T. gondii* which characterizes SEE, we performed a phylogenetic analysis of 67 strains isolated from and detected in nine species of intermediate hosts from both the domestic and sylvatic environment from Serbia.

## 2. Materials and Methods

### 2.1. Study Design

Genotyping was performed directly on strains detected in human fluids including amniotic fluid (AF), cerebrospinal fluid (CSF), peripheral or cord blood (PB, CB), and trypsin digested or mechanically homogenized animal tissues (heart, diaphragm) or after isolation by bioassay in Swiss Webster mice or short-term in vitro culture in VERO cells. Direct genotyping was preceded by gDNA concentration by Na-acetate/ethanol precipitation, while isolated gDNA was harvested from mouse brains after 1–2 sub-passages in vivo (3–6 months) or from infected VERO cell cultures (4–6 days).

### 2.2. T. gondii gDNA Detection

To detect the presence of *T. gondii* gDNA in the samples, the 529 bp repeat element (AF146527) was amplified using previously published protocol [22,23,24]. Briefly, each PCR reaction contained MaximaProbe Mastermix (Thermofisher Scientific, Waltham, MA, USA) or Taqman Universal Master Mix (Applied Biosystems, Foster City, CA, USA), specific forward and reverse primers (5′-AGA GAC ACC GGA ATG CGA TCT-3′; 3′-CCC TCT TCT CCA CTC TTC AAT TCT-5′), and TaqMan probe FAM-ACG CTT TCC TCG TGG TGA TGG CG-TAMRA (Invitrogen, Life Technologies, Carlsbad, CA, USA). The final reaction volume was 20 μL with 3 μL of extracted gDNA as template. After a hold interval of 2 min at 50 °C for UDG treatment and 10 min at 95 °C for polymerase activation according to the manufacturer’s instructions, the amplification was performed using 40–45 cycles of a two-step thermal profile. Fluorescence detection was performed during the annealing/extension step at 60 °C. Amplification and detection were performed in a StepOnePlus Real-Time PCR System (Applied Biosystems, Foster City, CA, USA).

### 2.3. Bioassay in Swiss Webster Mice for Strain Isolation

Strain isolation by bioassay was attempted from human blood, AF, and all animal tissues, except wild canid heart sections, which were frozen on receipt. The bioassay protocol was described in detail in the Refs. [25,26]. A maximum volume of a 500 µL suspension containing human fluids or trypsin-digested animal tissue and gentamycin (4 mg/kg, Galenika, Belgrade, Serbia) was injected into the peritoneum of Swiss Webster female mice weighing 18–20 g (*n* = 2 per human fluid sample or *n* = 2–4 per tissue digest). The mice were housed 2–4 per cage and were given water and food ad libitum and kept at a natural light cycle for 6 weeks until sacrifice. After 6 weeks, the mice were sacrificed by cervical dislocation, and the brains were removed and homogenized using an insulin syringe and a 21 G hypodermic needle in sterile saline (Hemofarm, Vršac, Serbia). A volume of 100 µL of the suspension was evenly divided on four glass slides and examined for the presence of tissue cysts by microscopy. The number of tissue cysts per brain was estimated as the total count on four slides ×10; a suspension of brain homogenate in sterile saline was then prepared which contained up to 20 tissue cysts/mL. A maximum of 500 µL of the suspension was administered to each mouse by oral gavage. All animals were monitored daily for clinical symptoms, and if symptoms such as a tottering gait or dyskinesia appeared during the first 14 days post-infection, they were treated with sulfadiazine (500 mg/L, Sigma-Aldrich, St. Louis, MO, USA) in drinking water for three consecutive days. Animals which developed severe symptoms at any time post-infection were euthanized to prevent unnecessary suffering. Strains were maintained by regular sub-passages in vivo at an interval of 3–6 months.

### 2.4. Nucleic Acids Extraction

Nucleic acids contained in human fluids were extracted using the DNeasy Blood and Tissue mini kit or the QIAmp DNA mini kit (Qiagen, Hilden, Germany) according to the manufacturer’s instructions. gDNA contained in animal tissue digests or organ homogenates was extracted using either the GeneJet Genomic DNA purification kit (Thermofisher Scientific, Waltham, MA, USA) according to the manufacturer’s instructions or using the Trizol Reagent (Invitrogen, Carlsbad, CA, USA).

### 2.5. DNA Precipitation by Na-Acetate/Ethanol

gDNA isolated from samples from which strain isolation was not possible (frozen canid heart tissue), unsuccessful, or not attempted was concentrated up to 20-fold by Na-acetate/ethanol precipitation. Briefly, 2 µL of a co-precipitant, PelletPaint (Merck-Millipore, Darmstadt, Germany), was added to each volume (20–100 µL) of gDNA to be concentrated along with 0.1 volumes of 3M Na-acetate (Merck-Millipore, Darmstadt, Germany) and mixed well. Next, two volumes of 100% ethanol were added to the sample and after a brief incubation at room temperature, the sample was centrifuged at top speed (16,000× *g*) in a tabletop centrifuge 5415R (Eppendorf, Hamburg, Germany) for 5 min. Next, the supernatant was discarded, the pellet was washed with 300 µL of 70% ethanol twice, and finally with 100% ethanol, and air-dried for 5–10 min. The pellet was re-suspended in a small volume of RNAse/DNAse free water.

### 2.6. Genotyping by MnPCR-RFLP and Genotype Identification

The MnPCR-RFLP method was performed as previously described by Su et al. [27] using nine loci: altSAG2, BTUB, GRA6, C-22, C-29, L358, PK1, Apico, and the additional locus CS3 described in the Refs. [16,28]. The multiplex reaction contained 1.5 mM of each external forward and reverse primer, Dreamtaq Green PCR Mastermix or PCR Mastermix (Thermofisher Scientific, Waltham, MA, USA), 2–3 µL of extracted gDNA, and DNAse-/RNAse-free water in a total volume of 20 µL. The nested reaction contained 3 mM of internal forward and reverse primers specific for a particular locus, Mastermix as in the multiplex reaction, and up to 2 µL of the multiplex reaction itself as a template. The reactions were performed in a Veriti thermal cycler (Applied Biosystems, Waltham, MA, USA). Reaction products were visualized on 1.5–2% agarose gels stained with EtBr. Each product was subsequently digested using the appropriate restriction enzymes as described in the Ref. [27], and the restriction fragments were visualized on 1.5% agarose gels stained with EtBr. Reference RH (ToxoDB#10), ME49 (ToxoDB#1), and NED (ToxoDB#2) strains were used as positive controls for each batch of MnPCR-RFLP and to identify allele-specific banding patterns at each locus. Genotypes were identified using the ToxoDB [www.toxodb.org (accessed on 20 June 2021)]. Genotypes were designated as recombinant if a mixture of two allele types was observed and the allele type at the Apico locus was different from the dominant allele type, while other differences from the archetypal genotypes were considered variants [29,30,31]. For the purposes of analysis, even partially obtained genotypes were classified in this manner.

### 2.7. Phylogenetic Analysis

The NJ tree was constructed using SplitsTree (https://software-ab.informatik.uni-tuebingen.de/download/splitstree4/welcome.html (accessed on 27 July 2021)). Strains were analyzed using all 10 standard genotyping loci according to the Ref. [27] and the additional locus CS3 [16,28]. The allele type on the CS3 locus for the reference genotypes was identified by sequence examination and conversion to the RFLP banding pattern, as described in the Ref. [32]. Included in the tree are only strains from Serbia which represent unique genotypes and have identified alleles on at least nine loci and the ToxoDB genotypes which are the closest match, as well as exemplary Brazilian strains published by the Ref. [16] (TgCatBr39, TgCatBr42, TgCatBr58, TgCatBr64), Spanish isolates (as representative for European strains) published by the Ref. [33] (TgPigSp2-ToxoDB#2, TgPigSp5-ToxoDB#3), and reference genotypes (GT1, ME49, VEG, MAS, TgCatBr5, CAST, FOU, P89, VAND, RUB, TgCtCo5, TgCgCa01/COUG).

## 3. Results

**Figure 1:**
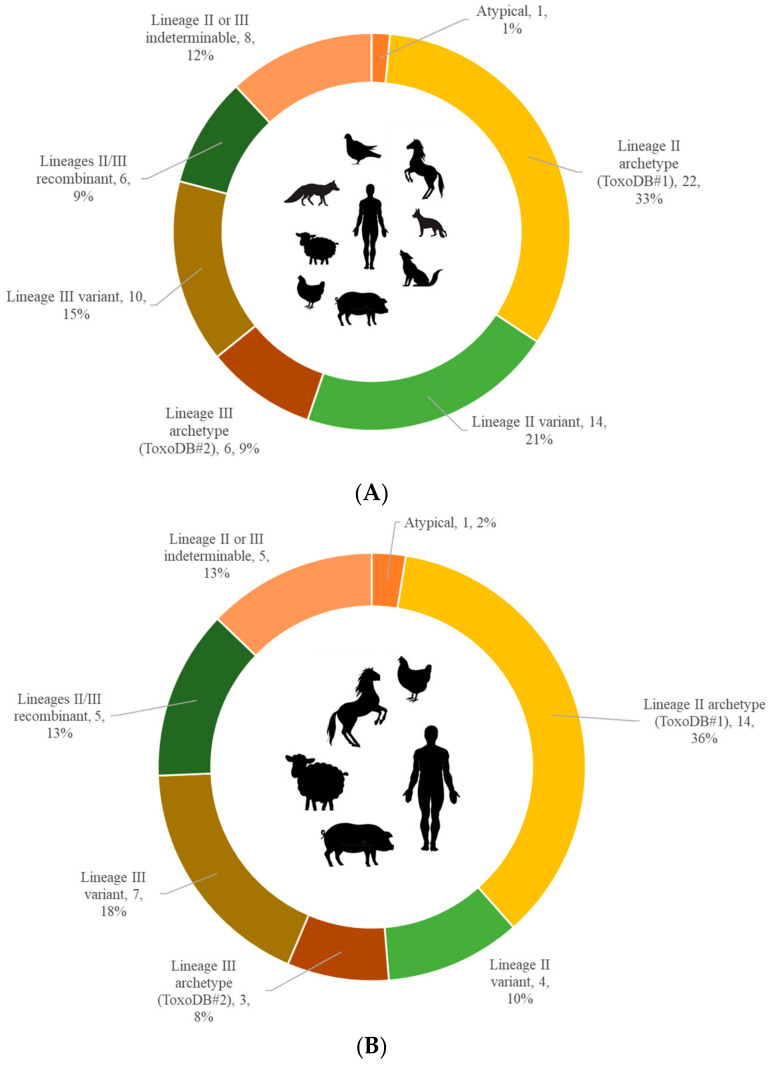

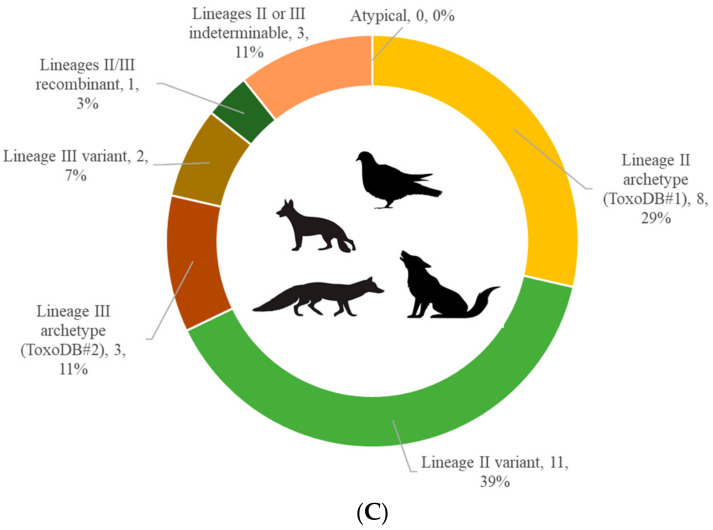
(**A**) Total population structure of *T. gondii* in intermediate hosts. The structure was inferred from 67 strains of human origin (*n* = 24), domestic animals (*n* = 15), and wildlife (*n* = 28), representing nine species of intermediate hosts; (**B**) Population structure of *T. gondii* in the domestic environment. The structure was inferred from 39 strains of human origin (*n* = 24) and domestic animals (*n* = 15), representing four species of intermediate hosts. The majority of strains detected in domestic pigs (*Sus scrofa domesticus*) originated from confined animals, while the strains isolated and detected in horses (*Equus caballus*), chicken (*Gallus gallus domesticus*), and sheep (*Ovis aries*) originated from free-range animals; (**C**) Population structure of *T. gondii* in the sylvatic environment. The structure was inferred from 28 strains from four species of wildlife—feral pigeons (*C. livia*, *n* = 3), golden jackals (*C. aureus*, *n* = 13), red foxes (*V. vulpes*, *n* = 10), and grey wolves (*C. lupus*, *n* = 2). Aside from the pigeons, who originated from areas in and around Belgrade, individual animals of the Canidae family originated from suburban, rural, and remote forested areas of Serbia. In (**A**–**C**), each genotype category is followed by the number of strains and percentage of the total structure.

In total, 67 strains from nine species of intermediate hosts were genotyped and used to infer the total population structure. Isolates (*n* = 12) were genotyped on nine loci, while direct genotyping from the source material was done for the remaining strains (*n* = 55), with four loci being the cutoff for inclusion into the population structure. Only four strains were genotyped on just four loci. Three strains from feral pigeons were included into the sylvatic structure, as the area from which the birds originated includes both the city of Belgrade and the surrounding suburbs. In total, 58.3% were strains from hosts residing in the domestic environment, while 41.7% were from hosts from the sylvatic environment. Overall, a little over half of the structure consisted of lineage II genotypes (54%), while lineage III genotypes accounted for nearly one quarter (24%). The lineage II archetype (ToxoDB#1) was the dominant genotype in intermediate hosts with 33% of the total strain population, followed by lineage II variant strains, with 21%. The lineage III archetype (ToxoDB#2) accounted for a mere 9%, and lineage III variant genotypes for 15%. The remaining 22% of the structure included genotypes which could be identified as recombinants of lineages II and III (9%), atypical genotypes (1%), and genotypes which could not be definitively classified into a particular lineage (12%) (Figure 1A).

Analysis according to the environment showed that genotypes which circulated in the domestic environment in humans and animals alike were also dominantly of lineage II with the archetype (ToxoDB#1) representing 36% of the structure, while variant genotypes accounted for only 10%. Lineage III made up 26% of the total structure, but in contrast to lineage II, the lineage III archetype (ToxoDB#2) made up 8%, while variant genotypes made up a significant 18%. Recombinant genotypes and those that could not be assigned to a lineage each made up 13%, and atypical genotypes 2% of the structure (Figure 1B).

In the wild environment, lineage II was dominant with even 68% of the structure, with the archetype (ToxoDB#1) accounting for 29% and variants being dominant with even 39% of the total structure. The archetype of lineage III (ToxoDB#2) represented 11% of the structure, while the variants only represented 7%. The remaining 14% consisted of recombinants and strains for which the lineage could not be precisely determined (Figure 1C).

Comparison of the MnPCR-RFLP results identified 31 distinct allele patterns, possibly indicating distinct genotypes (Table 1). Patterns 11 and 12 (strain IDs: 057-16, 061-16, 062-16, 075-16) which originated from golden jackals may represent the same genotype, which could not be matched in the ToxoDB. Patterns shaded blue (6, 7, 26) indicate genotypes which have been detected in hosts residing in both the domestic and sylvatic environment, while the rose-shaded patterns (3, 18) indicate genotypes which were detected in two host species from the domestic environment.

**Figure 2:**
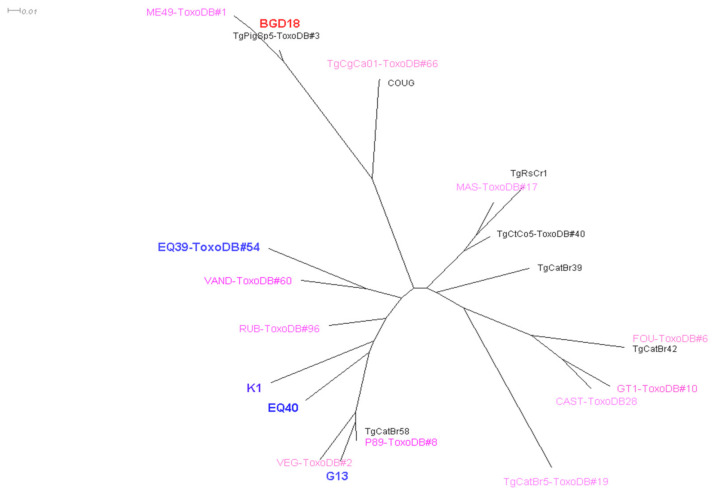
Neighbor-joining tree of *T. gondii* strains typed on a minimum of nine loci. Blue and red bold type indicates genotypes originating from Serbia, pink indicates reference strains, and black are isolates from Europe (TgPigSp5) and South America.

A NJ tree which included the Serbian isolates typed on nine markers was constructed (Figure 2). On the NJ tree, isolate EQ39 (ToxoDB#54) appears to be more related to ToxoDB#96 than TOXODB#2, while K1 and EQ40 are moderately related to ToxoDB#60—yet slightly more related to ToxoDB#96 than to ToxoDB#2 or TOXODB#8.

## 4. Discussion

Nearly half of the population structure inferred from 67 *T. gondii* strains from nine species of intermediate hosts consisted of variants and recombinants of lineages II and III (45%), while archetypes of both lineages represented 42%. The remaining 13% were strains which could not be assigned to a lineage because a full MnPCR-RFLP profile could not be obtained, and a single strain (TgH106019) determined as atypical at the time of detection by MS analysis [34]. This strain is interesting as it originated from an immunocompromised patient who reportedly never left the region, implying that the infection was acquired locally, suggesting that this genotype is possibly in circulation in SEE. However, recent re-evaluation of the MS marker lengths identified this genotype to be of lineage Africa 4, which was also detected in a case of congenital toxoplasmosis from Bulgaria [35], confirming that Africa 4 circulates in SEE. Along with the reports of Africa 1 genotypes in Turkey in humans as well as in animals [36,37], these findings indicate the presence of African lineages in SEE. Global distribution of Africa 1 has been suggested, which gains importance in view of the fact that Africa 1 has been shown to be virulent in mice; Africa 4, on the other hand, may have more limited distribution, and has been shown to be avirulent to mice [18]. No lineage I genotype was detected in Serbia. Lineage I is globally rare, despite reports showing that genotypes of this lineage do circulate in Europe [13], which could indicate that it is of lower frequency in SEE than in other parts of the continent [38,39].

In the domestic environment, ToxoDB#1 represented 36% of the strain population and was most frequent in pigs and humans (11/14). In fact, it represented 75% of the pig isolates and 100% of the human isolates (*n* = 5), with the remaining two pig isolates and only one strain detected in human AF being ToxoDB#2. Variants and recombinants, including genotypes which could not be assigned to a lineage but were clearly non-archetypal together represented more than half of the structure, and in fact, many of the variant genotypes were detected in but not isolated from human samples. Such a large representation of non-archetypal strains in human samples is not surprising, as a significant proportion variant and or recombinant genotypes (26%) was found in food animals. A certain diversity in human samples is expected and has been previously demonstrated in Europe and North America, that may be partly due to strain importation by travel and/or food globalization [40,41]. While successful strain isolation from human fluids is rather difficult primarily due to the usually low numbers of tachyzoites, it is of note that none of the variant genotypes could be isolated, while ToxoDB#1 was frequently isolated. It is tempting to speculate that there may be an additional bias in the isolate collection due to the inherent properties of tachyzoites of certain genotypes, such as ToxoDB#1, that allow enhanced host-to-host transmission.

In the sylvatic environment, lineage II variant genotypes (39%) were more frequent than ToxoDB#1 (29%). In fact, lineage II variants were also more common in the sylvatic than in the domestic environment. In contrast, lineage III variants represented 18% of the structure in the domestic environment, and a small share (7%) in the sylvatic structure. Recombinant genotypes also represented a very small share of the wildlife genotype population (3%), in contrast to their share in the domestic environment (13%), which may be due to a difference in the abundance of definitive hosts. *Felis silvestris*, which are distributed across the country, and *Lynx lynx*, which primarily inhabit forested areas distant from human settlements, are native to Serbia, yet the population size of both species is small and stands in stark contrast to the vast population size of domestic cats and the number of stray and pet cats with outdoor access [42,43]. Given this abundance of definitive hosts in the domestic environment, infections after oocyst ingestion, particularly in free-range animals, are expected to be frequent. In addition, both *F. silvestris* and *L. lynx* tend to avoid getting close to human settlements, unlike foxes and golden jackals, which indicates that these felids and canids do not share habitats, and could also explain the low number of recombinant genotypes detected in the canids [44,45].

The significant share of variant genotypes of lineages II and III in the total population structure and the likelihood that they represent 28 distinct genotypes indicates high diversity, which is somewhat surprising for Europe [13], yet may be due to the fact that strains from sylvatic hosts represented 41.7% of the total structure where a more diverse population of genotypes may be expected [14,19,20], and over half of the genotype population from the domestic environment originated from free-range animals. In fact, a recent report of atypical genotypes in wildlife from southern Italy supports the notion that the diversity of *T. gondii* in the sylvatic environment in southern Europe may be underestimated [46]. In addition, the bias of the population genetics data worldwide inherent to the sample collection strategy and isolation methodology greatly favors strains from food animals residing near human habitats. In light of that, there is a high probability of successful isolation of genotypes of predominantly low virulence to laboratory mice, which needs to be taken into account when interpreting the population structure and diversity in the genotype population circulating in a specific geographical area.

Interestingly, four genotypes (numbers 6, 7, 21, 22, 26 in Table 1) were detected in both the sylvatic and domestic environments, which is indicative of strain exchange between domestic animals, humans and wildlife. This is not unexpected, as habitats for many species of wildlife are fragmented and nestled in-between human settlements in Serbia and in fact most of Europe, while truly remote areas of unbroken, pristine wilderness are scarce.

Another interesting observation is that strains 061-13 detected in a red fox, and K1 isolated from a free-range chicken raised in close proximity to peri-urban hunting grounds, were found to be of identical genotype; we have previously determined, based on cumulative mortality of Swiss Webster mice (38.8%), that this genotype is intermediately virulent [23]. This finding could be of concern as, in fact, many genotypes which circulate in the sylvatic ecosystem have been found to cause serious clinical presentations and even lead to death in people in different parts of the world [47,48,49]. In light of the habitat distribution of wildlife in Serbia, it is evident that more virulent genotypes can be expected in both environments.

The diversity of lineage III in the genotype population in the domestic environment in Serbia is notable. This lineage is thought to be ancestral to Africa, and a significant presence of lineage III variants in humans and domestic animals has not been widely reported in Europe [18,50]. In fact, the intermediately virulent genotype EQ40 is likely endemic in southern Europe, as it has been previously isolated in Portugal and France, but it may have been imported originally from Africa, as the MS marker lengths match a strain from Gabon—over 5000 km away [18,23,50,51]. The intermediately virulent chicken isolate K1 and EQ40 may be more related to ToxoDB#96 than to ToxoDB#2. The other genotype isolated from a horse in Serbia, EQ39 (ToxoDB#54), which is of low virulence, has been detected in several animal species in the U.S.—nearly 9000 km away [52], and appears to be moderately related to ToxoDB#60.

Given the evolutionary history of *T. gondii*, the worldwide presence of genotypes of African origin and/or of those closely related to them, is not surprising [18,53,54]. It is likely that strain exchange between Europe and Africa facilitated by recurrent and/or historical animal migrations led to the gradual accumulation of *T. gondii* genotypes of African origin in southern Europe—with recurrent migrations probably being of far greater consequence in terms of the current diversity of the genotype population in SEE. There are several major migratory bird flyways crossing the Mediterranean and southern European countries act as stopover points for vast numbers of birds, estimated at over 2 billion [55], en route to and from Africa. This provides an opportunity, while the fact that they may spend a considerable amount of time on the ground depending on the level of energy reserves [56], exhaustion, and/or possible sickness may increase the probability of predation at the resting grounds, and thus, strain introduction into a new geographical environment. In addition, the golden jackal, a migratory species which is rapidly expanding its range [57,58], with large resident populations in SEE and vagrant populations in other areas of southern Europe, was shown to be a major reservoir for different parasite species, including both *Trichinella britovi* and *Trichinella spiralis*, and *T. gondii*, and may be an instrumental vector for the distribution of genotypes across the continent [58,59].

It is difficult to estimate whether EQ40 and EQ39 (ToxoDB#54) are recent or historical exports from Africa to Europe and Europe to North America, respectively, but their vast geographical distribution and findings in different host species favor the latter. Given the different degrees of virulence, however, the supposed evolutionary trade-off between virulence and transmission, which may explain the low frequency of the virulent lineage I worldwide, is difficult to discern [60,61]. Clearly, virulent genotypes must possess adaptations to allow for increased transmission capacity in order to persist evolutionarily. Yet, this would imply that a number of the more virulent genotypes detected in SEE may persist along with the multitude of genotypes of low virulence. The findings of virulent genotypes in domestic animals in SEE along with the reports from Spain [23,62], where mouse virulent genotypes have also been isolated from pigs and sheep, call for vigilance as the impact of infection with more virulent genotypes may be underappreciated in Europe.

## Figures and Tables

**Table 1:** Distinct *T. gondii* genotypes detected and/or isolated in intermediate hosts from Serbia.

						Genotyping Loci									
				*Chromosome*		*VIII*	*IX*	*X*	*Ib*	*Plastid*	*III*	*V*	*VI*	*VIIa*	
Number	Origin	Isolate	Tissue	Lineage	Internal strain ID	AltSAG2	BTUB	GRA6	C22-8	Apico	C29-2	L358	PK1	CS3	ToxoDB genotype/isolate ID
1	Multiple species	N	Blood, AF, Heart	III ARCH	various	III	III	III	III	III	III	III	III	III	ToxoDB#2
2	Multiple species	Y	AF, Heart	II ARCH	various	II	II	II	II	II	II	II	II	II	ToxoDB#1
3	Horse, human	Y	Heart	II/III, rec	EQ39, 123c20	II *	III	III *	III	II	III	III	III *	II *	ToxoDB#54
4	Horse	Y	Heart	III VAR	EQ40	II	I	III	II	III	III	III	II	III	TgPiPr14
5	Pigeon	Y	Heart	III VAR	G13	III	III	III	II	III	III	III	II	III	No match
6	Chicken, red fox	Y	Heart	III VAR	K1, 061-13	II *	II *	I *	II *	III	III	III	III *	III *	No match
7	Golden jackal, human	N	Heart, AF	II/III, rec	125-16, 46c21	II *	II *	II *	II	III *	-	-	III *	II	No match
8	Human	N	AF	II or III VAR	25c19, 100c16	III *	III *	II *	II	III	-	-	I *	II *	No match
9	Human	N	AF	III VAR	182c19	III	III	III	I	III		-	III	II	ToxoDB#21, #120, #123 (w/o CS3)
10	Golden jackal	N	heart	II VAR	051-16	II	II	II	III	-	-	-	III	I	No match
11	Golden jackal	N	Heart	II VAR	057-16, 061-16, 062-16	II *	III *	III *	II *	II	-	-	II *	-	No match
12	Golden jackal	N	Heart	II or III	075-16, 061-16, 062-16	II *	III *	III *	II *	-	-	-	II *	III	No match
13	Red fox	N	Heart	II VAR	068-16	II	III	II	II	II	-	-	III	-	No match
14	Red fox, grey wolf	N	Heart	II VAR	103-16	II *	III *	II *	II *	-	-	-	II *	II	No match
15	Grey wolf	N	Heart	II or III	020-16	II	III	II	II	III	-	-	III	-	No match
16	Human	N	AF	II VAR	107AF	II	II	II	I	-	-	-	III	II	No match
17	Human	N	AF	II VAR	8c21	II	-	II	III	III	-	-	III	II	No match
18	Horse, human	N	Heart	II/III, rec	EQ12, BAL	II *	II *	III *	-	III	-	-	II *	II	No match
19	Human	N	AF	III VAR	138c18	III	-	III	II	III	-	-	III	II	ToxoDB#116, #130, #163 #187 (w/o CS3)
20	Human	N	AF	III VAR	108c16	III	III	II	-	III	-	-	II	II	No match
21	Red fox, human	N	Heart	II VAR	113-16, 158c18	II	III	II *	-	-	-	-	I *	II *	ToxoDB#45, #142, #149, #150, #203 (w/o CS3)
22	Red fox, human	N	Heart, AF	III VAR	124-16, 82c16	III *	II *	III *	III	-	-	-	III *	-	No match
23	Golden jackal	N	Heart	II VAR	023-15	II	III	II	II	-	-	-	III	-	No match
24	Human	N	CSF	II VAR	269L	III	II	III	II	-	-	-	II	-	No match
25	Human	N	AF	III VAR	132c15	III	-	III	-	III	-	-	II	II	No match
26	Human, red fox	N	AF	II or III	97c18, 121-16	II *	III *	II *	-	-	-	-	III	II	ToxoDB#136 (all markers), #93, #105, #107, #135, #136, #137, #139, #172, #173, #174, #202 (w/o CS3)
27	Human	N	AF	III VAR	83c16	III	II	II	III	-	-	-	III	-	No match
28	Human	N	AF	III VAR	65c20	III	-	I	III	II	-	-	III	-	No match

* Indicates matching alleles; AF, amniotic fluid.

## Data Availability

The data presented in this study are available on request from the corresponding author.
